# Supplementary material for: Mechanisms of site‐specific dephosphorylation and kinase opposition imposed by PP2A regulatory subunits
Source: EMBO J. 2020 May 13;39(13):e103695. doi: 10.15252/embj.2019103695 (PMC7327492; doi:10.15252/embj.2019103695)
Supplement: Supplementary file 1 — Expanded View Figures PDF [file EMBJ-39-e103695-s001.pdf]

Expanded View Figures

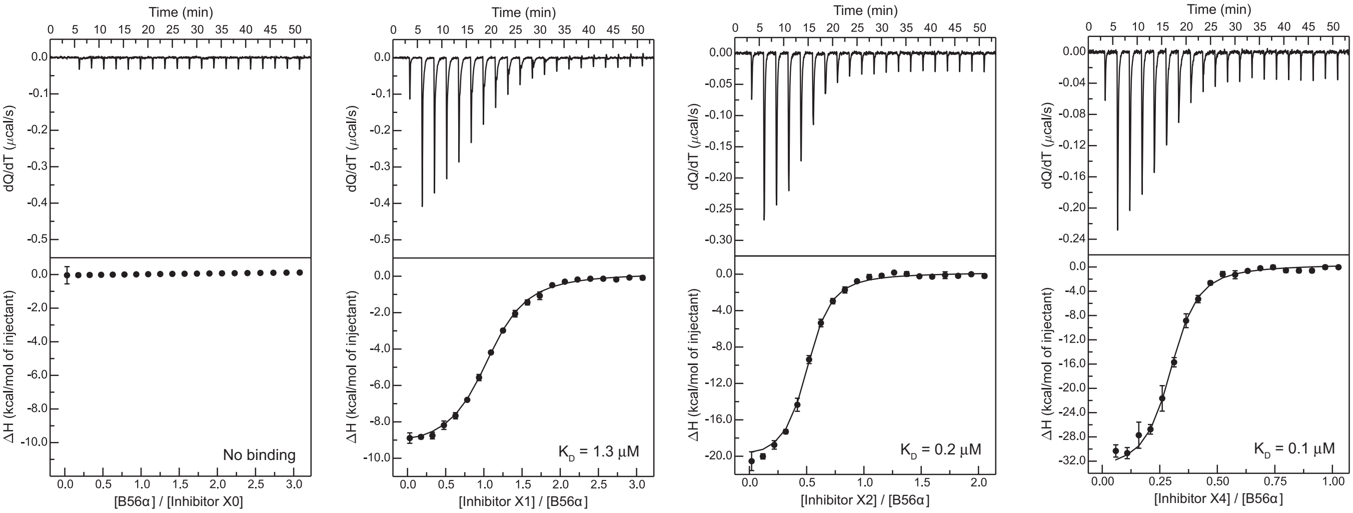

Figure EV1. Binding affinity of B56 inhibitors to recombinant B56.

A ITC measurements using B56α and indicated His-tag versions of ctrl and B56 inhibitors.

Figure EV2. Phosphoproteomics on cells expressing the B56 inhibitor.

A Live-cell imaging of cells expressing doxycycline-inducible B56 inhibitor. Cell were released from a thymidine block and followed into mitosis. Doxycycline was added at time 0 h.

B Quantification of mitotic duration of cells from (A) Each circle represents a single cell and median time in mitosis indicated by red line. A representative result from at least three independent experiments is shown. At least 20 cells were counted per condition in the experiment shown. NEBD; nuclear envelope breakdown.

C Comparison of log_2 ratios of B56-dependent dephosphorylation sites versus other phosphorylation sites on the same protein.

D IceLogo representation of over- and underrepresented amino acid residues surrounding phosphorylation sites for the down-regulated phosphorylation sites from experiments presented in Fig 2A and B.

E Venn diagram showing overlap of PP2A-B56-regulated sites in G1/S and mitosis (M).

F Schematic of *in vitro* peptide phosphorylation assay set-up corresponding to IceLogo of PP2A-B56-regulated sites in Fig 2F.

G Schematic of LxxIxE inhibition experiment in mitotic lysate corresponding to IceLogo of PP2A-B56-regulated sites in Fig 2G.

H Mitotic cell lysates treated with thiophosphorylated wt or S62A GST-Arrp19 for 5 min followed by Western blotting with the antibodies indicated.

Source data are available online for this figure.

© The Authors

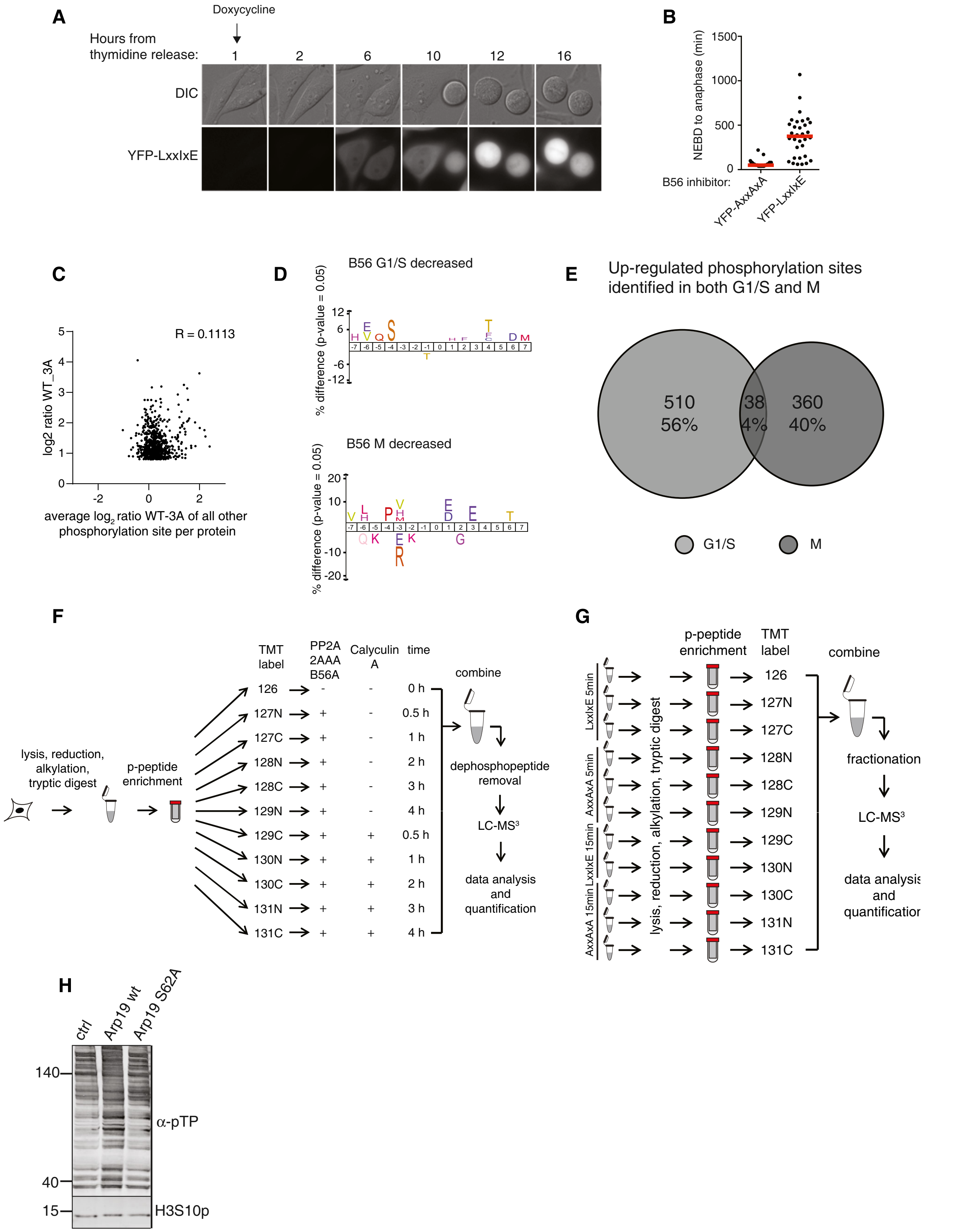

Figure EV2.

 © The Authors

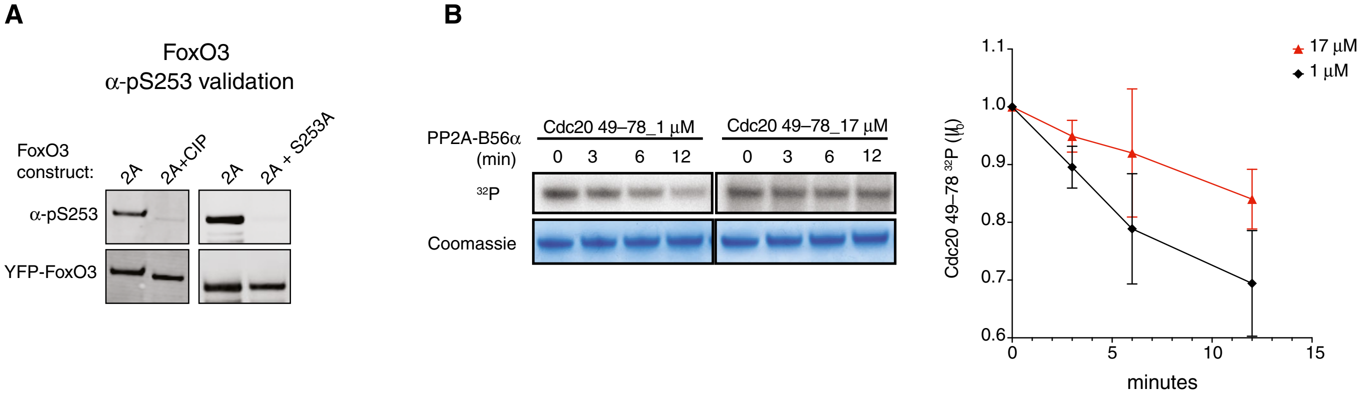

Figure EV3. Dephosphorylation of FoxO3 by PP2A-B56.

A Validation of FoxO3 α-pS253 antibody. The indicated YFP-FoxO3 variants were immunopurified and subjected to Western blotting with the indicated antibodies. CIP; calf intestinal phosphatase, 2A; FoxO3 M448A I451A unable to bind PP2A-B56.

B Dephosphorylation by the PP2A-B56α holoenzyme complex of GST-Cdc20 49–78 substrates with LxxIxE motifs of different binding affinities for B56 as indicated. The mean and standard deviation of 3 independent experiments are shown.

Source data are available online for this figure.

Figure EV4. Regulation of ADAM17 by PP2A-B56.

A ITC measurements of the binding of B56α to the indicated ADAM17 peptides.

B *In vitro* phosphorylation of GST and GST-ADAM17 with PKA.

C Western blot of ADAM17 expression in the parental wild-type (wt) DLD-1 cell line and ADAM17 knockout (ADAM17$^{-/-}$) clones #1 and #2.

D Sanger sequences of the edited coding region in *ADAM17* of DLD-1 wt and ADAM17$^{-/-}$ clones #1 and #2.

E Re-expression of ADAM17 in ADAM17$^{-/-}$ (clone #1) cells, determined by Western blot.

F Amphiregulin (AREG) shedding measured by ELISA of conditioned media from ADAM17$^{-/-}$ cells (clone #1) with or without ADAM17 re-expression (A17$^{-/-}$+A17). Two-sided, unpaired Student's *t*-test was applied to test for significant differences $^{**}P < 0.01$. Mean and standard deviation indicated from three independent experiments.

G Western blot of ADAM17 in streptavidin pull-downs (PD: Strep) and total cell lysates (TCL) from cell surface biotinylated DLD-1 ADAM17$^{-/-}$ cells (clone #1) re-expressing ADAM17 variants (wt, I761 or LEE).

H Amphiregulin (AREG) shedding measured by ELISA in conditioned media from untreated, H_2O_2 treated, or radiated DLD-1 ADAM17$^{-/-}$ cells (clone #2) re-expressing ADAM17 variants (wt, I761 or LEE). Two-sided, unpaired Student's *t*-test was applied to test for significant differences $^{*}P < 0.05$, $^{**}P < 0.01$ and $^{***}P < 0.001$. Mean and standard deviation indicated from at least three independent experiments.

I Western blot of ADAM17 expression in the parental 4T1 cells line (wt) and 4T1 ADAM17 knockout clone (A17$^{-/-}$).

J Sanger sequences of the edited coding region in *Adam17* of the 4T1 wt and A17$^{-/-}$ cell lines. GAPDH was used as an internal loading control in all Western blots. Shedding data represent average values ± SEM of at least 3 independent experiments.

Source data are available online for this figure.

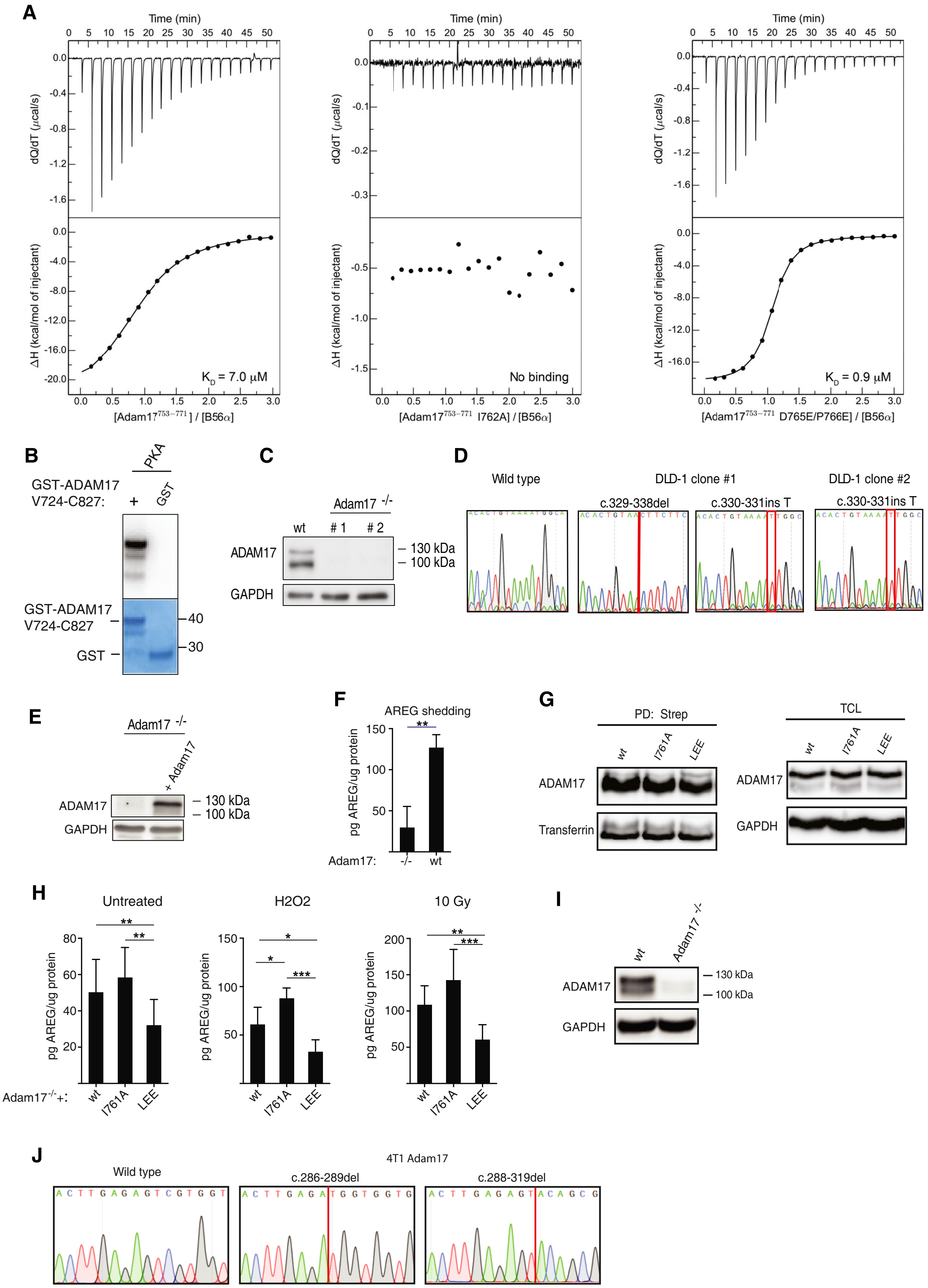

Figure EV4.

© The Authors
