# Supplementary material for: Mechanisms of site‐specific dephosphorylation and kinase opposition imposed by PP2A regulatory subunits
Source: EMBO J. 2020 May 13;39(13):e103695. doi: 10.15252/embj.2019103695 (PMC7327492; doi:10.15252/embj.2019103695)

Figure EV3A

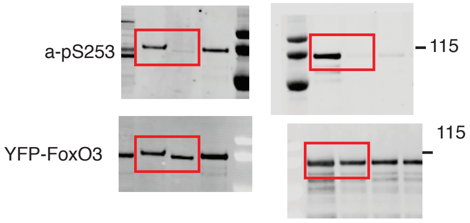

Figure EV3B:

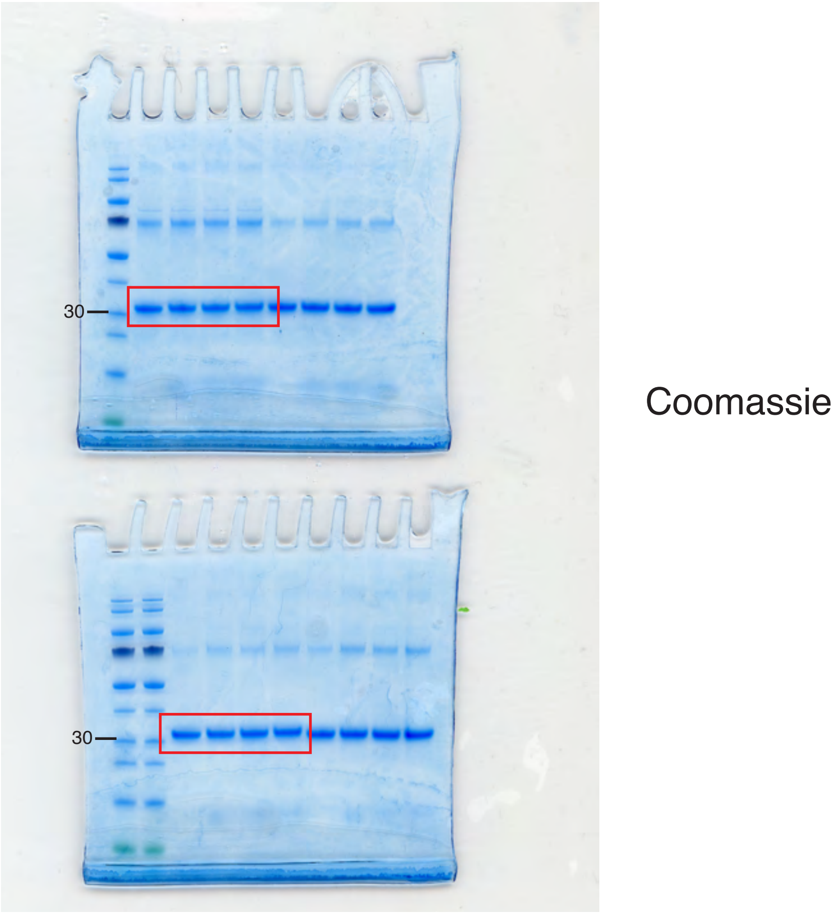

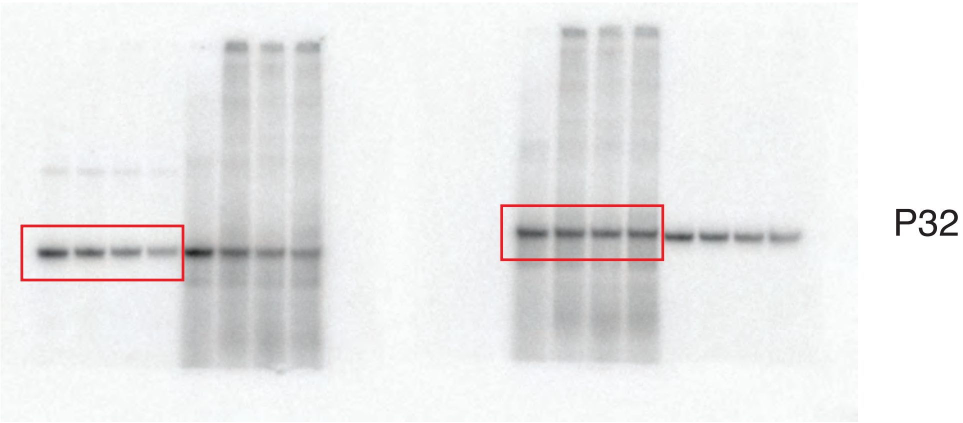

Supplement: Supplementary file 9 — Source Data for Expanded View [file EMBJ-39-e103695-s009.zip › embj2019103695-sup-0010-SDataFigEV3.zip!/EMBOJ-2019-103695R1-Figure_EV3_Source_Data-sd.pdf]
