# Supplementary material for: Mechanisms of site‐specific dephosphorylation and kinase opposition imposed by PP2A regulatory subunits
Source: EMBO J. 2020 May 13;39(13):e103695. doi: 10.15252/embj.2019103695 (PMC7327492; doi:10.15252/embj.2019103695)

Figure EV4C

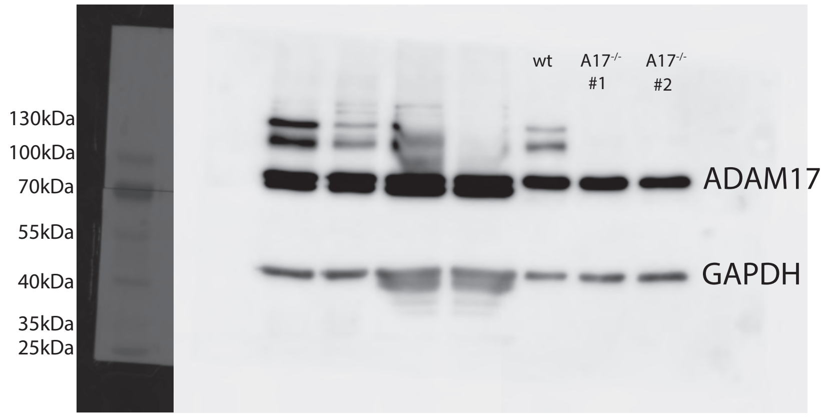

Figure EV4I

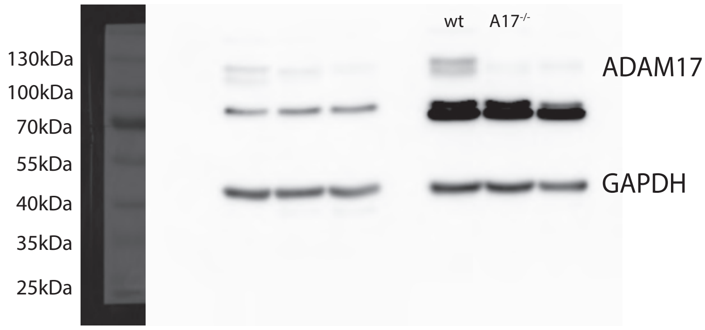

Figure EV4E

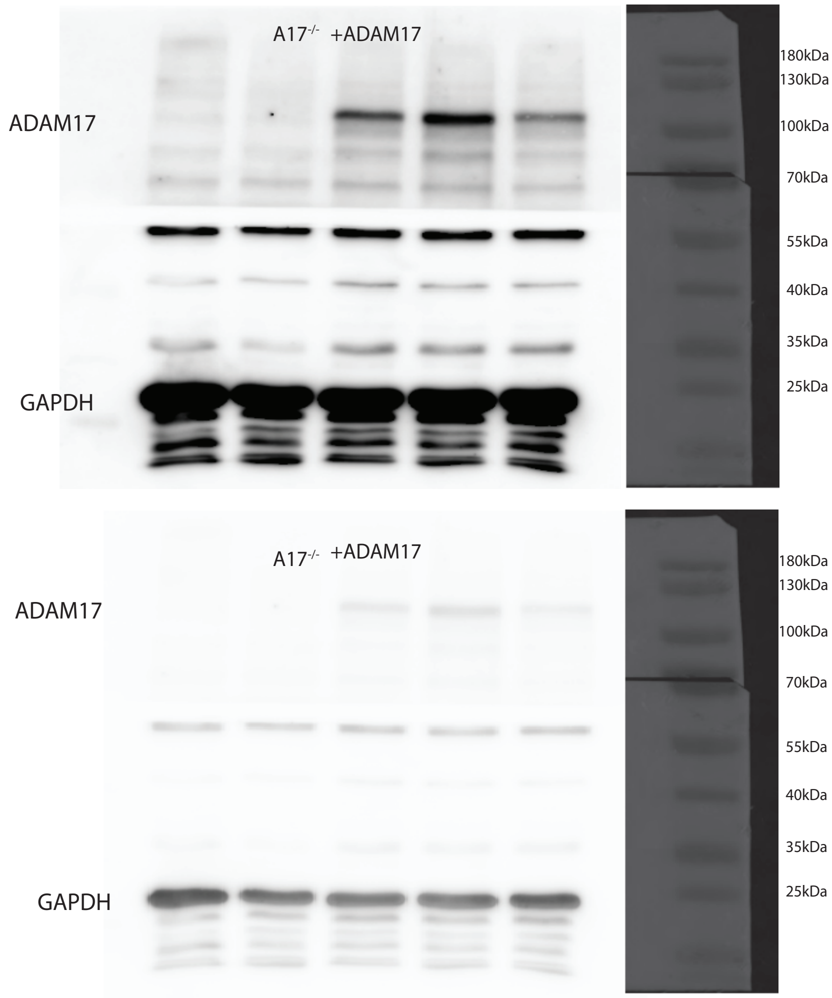

Figure EV4B

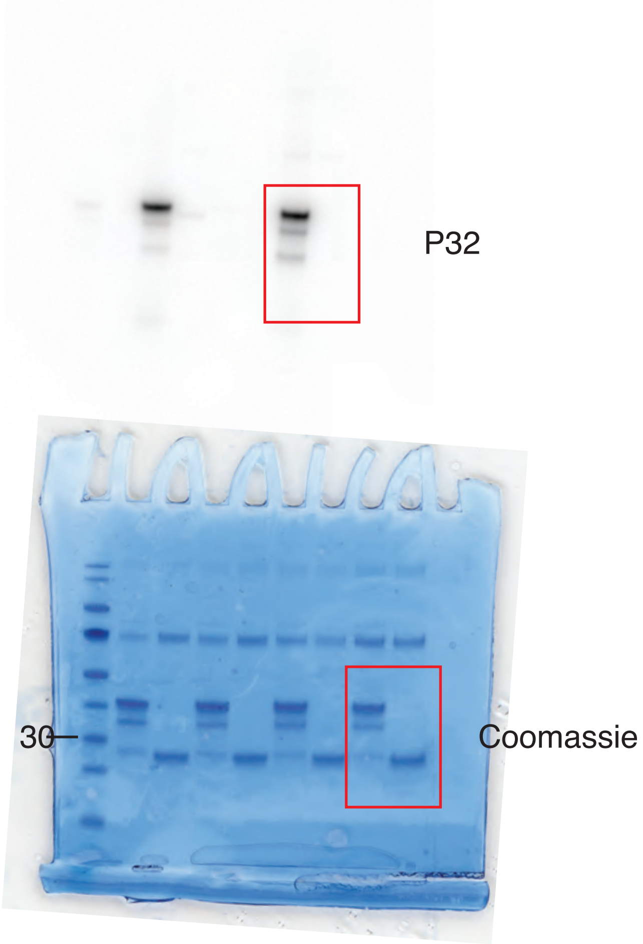

Figure EV4G

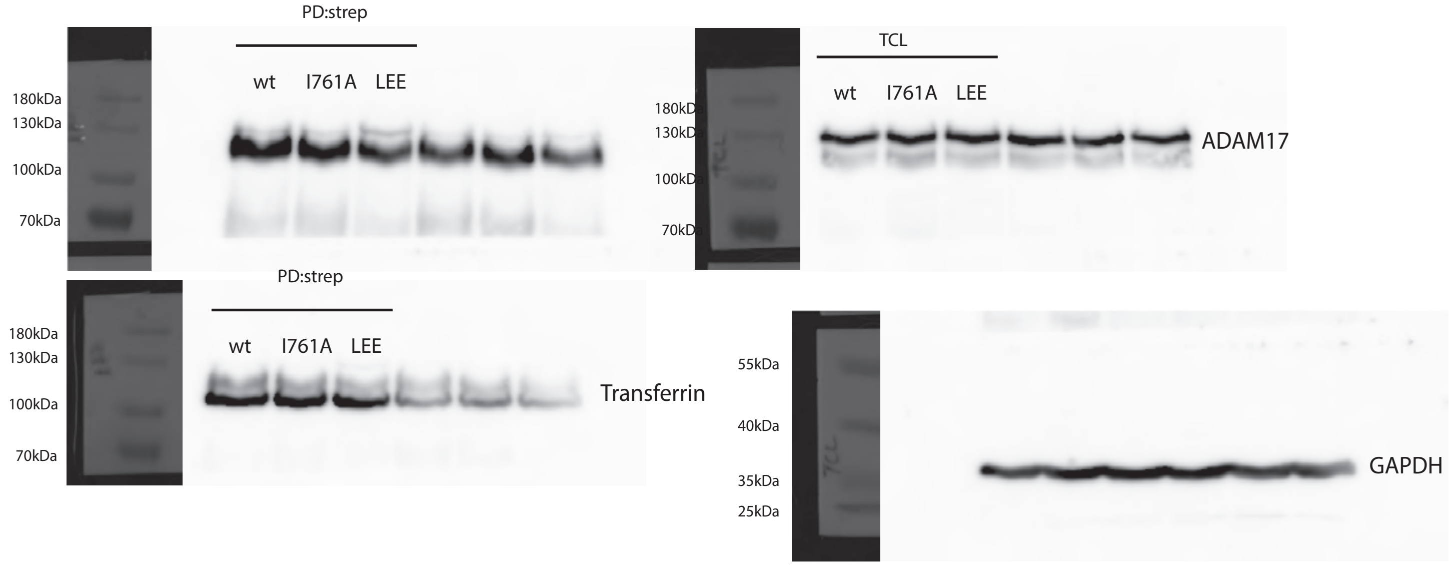

Supplement: Supplementary file 9 — Source Data for Expanded View [file EMBJ-39-e103695-s009.zip › embj2019103695-sup-0011-SDataFigEV4.zip!/EMBOJ-2019-103695R1-Figure_EV4_Source_Data-sd.pdf]
